# Mind the gaps - the epidemiology of poor-quality anti-malarials in the malarious world - analysis of the WorldWide Antimalarial Resistance Network database

**DOI:** 10.1186/1475-2875-13-139

**Published:** 2014-04-08

**Authors:** Patricia Tabernero, Facundo M Fernández, Michael Green, Philippe J Guerin, Paul N Newton

**Affiliations:** 1 Worldwide Antimalarial Resistance Network (WWARN), Churchill Hospital, University of Oxford, Oxford, UK; 2 Centre for Tropical Medicine, Nuffield Department of Clinical Medicine, Churchill Hospital, University of Oxford, Oxford, UK; 3 Lao-Oxford-Mahosot Hospital-Wellcome Trust Research Unit (LOMWRU), Microbiology Laboratory, Mahosot Hospital, Vientiane, Lao PDR; 4 School of Chemistry & Biochemistry, Georgia Institute of Technology, Atlanta, GA, USA; 5 Division of Parasitic Diseases and Malaria, US Centre for Disease Control and Prevention, Atlanta, GA, USA; 6 London School of Hygiene and Tropical Medicine, London, England, UK

## Abstract

**Background:**

Poor quality medicines threaten the lives of millions of patients and are alarmingly common in many parts of the world. Nevertheless, the global extent of the problem remains unknown. Accurate estimates of the epidemiology of poor quality medicines are sparse and are influenced by sampling methodology and diverse chemical analysis techniques. In order to understand the existing data, the Antimalarial Quality Scientific Group at WWARN built a comprehensive, open-access, global database and linked Antimalarial Quality Surveyor, an online visualization tool. Analysis of the database is described here, the limitations of the studies and data reported, and their public health implications discussed.

**Methods:**

The database collates customized summaries of 251 published anti-malarial quality reports in English, French and Spanish by time and location since 1946. It also includes information on assays to determine quality, sampling and medicine regulation.

**Results:**

No publicly available reports for 60.6% (63) of the 104 malaria-endemic countries were found. Out of 9,348 anti-malarials sampled, 30.1% (2,813) failed chemical/packaging quality tests with 39.3% classified as falsified, 2.3% as substandard and 58.3% as poor quality without evidence available to categorize them as either substandard or falsified. Only 32.3% of the reports explicitly described their definitions of medicine quality and just 9.1% (855) of the samples collected in 4.6% (six) surveys were conducted using random sampling techniques. Packaging analysis was only described in 21.5% of publications and up to twenty wrong active ingredients were found in falsified anti-malarials.

**Conclusions:**

There are severe neglected problems with anti-malarial quality but there are important caveats to accurately estimate the prevalence and distribution of poor quality anti-malarials. The lack of reports in many malaria-endemic areas, inadequate sampling techniques and inadequate chemical analytical methods and instrumental procedures emphasizes the need to interpret medicine quality results with caution. The available evidence demonstrates the need for more investment to improve both sampling and analytical methodology and to achieve consensus in defining different types of poor quality medicines.

## Background

Malaria, in the world’s 104 endemic countries, occurs predominantly among intertwined poverty and lack of access to efficacious medicines [1]. A grossly neglected aspect of malaria control is the importance that patients should not just have access to medicines, but should have access to good quality-assured medicines. This was recently emphasized in the resolution adopted by the United Nations Human Rights Council “Access to medicines in the context of the right of everyone to the enjoyment of the highest attainable standard of physical and mental health, to medicines that are affordable, safe, efficacious and of quality” [2].

In the last decade many studies have highlighted deficiencies in medicine quality afflicting all classes of medicines, with no country immune [3-11]. The problem is not new, with reports of falsified cinchona bark from the 1600s [12] and falsified quinine from the 1800s [13-15].

There has been considerable confusion over definitions of different types of poor quality medicines [16-18]. The terms falsified (i.e. produced by criminals fraudulently), substandard (i.e. unintentional but negligent errors in factory processes) and degraded (i.e. degradation through inadequate storage after leaving the factory or interaction with inadequate excipients) were used to define medicine quality. With increasing use of the term ‘counterfeit’ to refer to intellectual property (IP) concerns, the term falsified was used to avoid IP associations. Public health should be the prime consideration in defining and combating poor quality medicines [19,20]. Nonetheless, there remains a lack of worldwide consensus on what constitutes a falsified medicine and no international treaty to criminalize the manufacturer or distributor of falsified medicines [16].

Falsified medicines may usually be identified by their fake packaging but both packaging and chemical analysis are required to be sure of a sample’s regulatory status and public health impact. Such investigations are difficult as instrumental and chemical analysis with sophisticated and expensive equipment, reagents and technical capacity are essential. For packaging analysis, genuine examples of products, direct from the manufacturer, are needed as comparators but are difficult to obtain [5]. Recently, portable instruments to determine the quality of anti-malarials, such as Raman spectrometers and the Global Pharma Health Fund GPHF*-*Minilab*®*, have become available and are being used to screen medicine chemical quality [21-23]. For forensic investigations, innovative chemical fingerprinting and palynology techniques can provide clues as to the origin of medicines through analysis of their mineral and pollen composition, respectively [5,24-26].

The consequences of using poor quality medicines range from prolonged sickness, treatment failure, side effects, loss of income, increased healthcare costs and death. In addition, societies may lose confidence in otherwise effective medicines, in healthcare systems and suffer major economic losses. Of particular current relevance, falsified or substandard anti-malarials containing subtherapeutic amounts of artemisinin derivatives or only one of the two active ingredients in artemisinin combination therapy (ACT), the primary treatment recommended for uncomplicated falciparum malaria, are very likely to contribute to disastrous anti-malarial artemisinin resistance [27,28], increasing mortality and morbidity and risking the loss of these vital medicines for malaria control.

Nevertheless, objective data on the geography and epidemiology of poor quality medicines are sparse. Estimates of anti-malarial quality vary widely depending on the sampling methodology used, with most reports not employing rigorous scientific techniques, potentially biasing results [29]. In order to understand the shortcomings of the data and how the existing data may help inform policy to improve anti-malarial quality, an openly accessible databank of all the published reports of the quality of anti-malarials was developed: the WWARN Antimalarial Quality Surveyor [9,30]. Here, the Surveyor is described, the database analysed and the implications for public health and potential interventions discussed.

## Methods

### Structure of the Antimalarial Quality Surveyor Database

A systematic review was conducted of scientific and lay reports on anti-malarial medicine quality, using Pubmed, Scielo, Embase, Embase-Classic, Medline, Google, Google Scholar, World Health Organization (WHO), United States Pharmacopeia (USP), and Medicines Regulatory Agencies (MRA) websites from 1946 to March 2013 in English, French and Spanish (Table 1). Search terms used were ‘anti-malarials’ or ‘artemisinin derivatives’ or ‘antimalarial agent’ and ‘counterfeit’ or ‘substandard’ or ‘fake’ or ‘spurious’ or ‘falsified’ or ‘quality’. Abstracts and full text of 404 studies were reviewed (See the “Inclusion and exclusion criteria of published reports included in WWARN database” subsection; Additional file 1). Data were extracted and entered in a database constructed using MS Access 2007. Descriptive analysis was conducted in Excel and STATA (v11.2, Stata Corp, College Station, TX, USA).

**Table 1:** Websites used for information searching about poor quality medicines

**International Organisations and NGOs**	**Medicine Regulatory Authorities and national bodies**	**Pharmaceutical Industry**
WHO Essential Medicines and Pharmaceutical Policies	Ghana Food and Drugs Board	Securing Pharma
WHO Prequalification Programme	Kenya Pharmacy and Poisons Board	Pharmaceutical Security Institute
International Medical Products Anti-Counterfeiting Taskforce (IMPACT)	NAFDAC Nigeria	Reconnaissance International
International Conference of Drug Regulatory Authorities	Thailand Food and Drugs Administration	No to Fakes
ReMeD-Réseau Médicaments et Développement	Health Sciences Authority, Government of Singapore	Sanofi
Fondation Chirac	Medicines and Healthcare products Regulatory Agency, UK Government	Pfizer
Medical Products Counterfeiting and Pharmaceutical Crime (MPCPC) Unit of INTERPOL	US Food and Drug Administration	
Permanent Forum on International Pharmaceutical Crime	Centres for Disease Control and Prevention, USA. Counterfeit and Substandard Antimalarial Drugs	**Alert lists and systems**
Medicines Transparency Alliance		WHO-WPRO Rapid Alert System for Counterfeit Medicines
Médecins Sans Frontières Campaign for Access to Essential Medicines	**Academic/Research Initiatives**	E-drug
Third World Network	ACT Consortium	E-med
Council of Europe-Medicrime convention	ACT Watch	E-fármacos
European Alliance to safe meds	QUAMED, ITM, Antwerp	Partnership for safe medicines
United Nations Office for Drugs and Crime	Counterfeit Drug Forensic Investigation Network (CODFIN)	Mpedigree
Thai Pharmaceutical System Research and Development Foundation (PhaRed)	Institut de Recherche sur l'Asie du Sud-Est Contemporaine	PharmaSecure
United States Pharmacopeial Convention (USP) PQM: Promoting the Quality of Medicines in Developing Countries	Chatham House	Sproxil
The Global Pharma Health Fund (GPHF)	Africa fighting Malaria	Safe Medicines Beta
IRACM Institute of Research Against Counterfeit Medicines	The CONPHIRMER consortium	Pharmabiz
	ReAct	
**Newspaper websites with interest in medicine quality**		
http://www.ghanaweb.com	http://www.allafrica.com	
http://www.modernghana.com	http://www.afrolnews.com	
http://www.africasia.com	http://www.tribune.com.ng/index.php	
http://www.monitor.co.ug	http://www.newtimes.com.gh/	
		

### Inclusion and exclusion criteria of published reports included in WWARN database

Inclusion criteria; Any of:

– Any study describing in vivo or in vitro tests to determine anti-malarial medicine quality, assays to determine quality, discussion over sampling methodology & pharmaceutical legislation

– Any published report from 1946 to March 2013 in English, French and Spanish

– Articles about seizures, recalls and confiscations of anti-malarials

– Case reports or articles describing side effects or patients not responding to anti-malarial treatments where quality was questioned

– Studies with results from several countries or locations are included under each specific country/location.

Exclusion criteria:

– Studies with results for a whole region or a whole class of drugs, without specific country or location data.

### Key variables and definitions

Anti-malarial quality failure rate is quoted from each report without additional analysis. If additional important information, other than packaging and amount of active pharmaceutical ingredient (API), such as disintegration, dissolution, and microbiology are given they are also included in the database. (For a more detailed description of each variable and methodology see [31]).

In view of the controversy over the terms used to describe medicine quality, author’s definitions were tabulated in and interpreted relation to the definitions used by WHO (Additional file 2), whilst a consensus is sought. ‘Falsified’ is used as a synonym for counterfeit or spurious, referring to a medical product produced with criminal intent to mislead, but without reference to intellectual property concerns. Samples that failed chemical assays, but without detection of wrong active ingredients and without packaging analysis, are classified as poor quality and not as falsified or substandard as this distinction cannot be reliably made without reference to the packaging [17]. However, samples that contained wrong API or no API but without packaging analysis were assumed to be falsified. There is a small risk of misclassification of such samples as falsified when they are actually substandard, due to gross manufacturing errors. However, such catastrophic errors of potential criminal negligence appear to be relatively rare [6,7]. Samples that did not fail chemical and packaging tests (when these were done) are considered as good quality. As there is little information available to distinguish substandard (i.e. errors in factory production) from degraded (i.e. degradation due to post-production inappropriate storage) medicines, substandard medicines may also, in error, include degraded products that left the factory as good quality [17,32].

### Antimalarial Quality Surveyor

The Antimalarial Quality (AQ) Surveyor is an open access, web-based visualization tool that tabulates and maps published reports on the quality of anti-malarial medicines [9]. The system was designed with medicine regulatory authorities (MRAs), national malaria control programmes (NMCPs) and medicine funding agencies as the target main users. A simple dashboard allows users to filter and explore the data and examine standardized summaries of anti-malarial quality reports. Key information that can be filtered includes the quality of anti-malarial medicines, where they were obtained and from what type of outlet and what sampling techniques and chemical assays were used.

The AQ Surveyor also includes a filterable tabular view, listing all the source reports plus other publications, such as reviews and descriptions of assay techniques, without primary data amenable to mapping, but relevant to medicine quality. The AQ Surveyor was reviewed by key audiences, including MRAs, NMCPs, pharmacists, and academics for feedback and adapted accordingly.

## Results

Of the 404 reports reviewed, 251 were eligible for inclusion into the database (Additional file 1). Of publications included, 51.8% (130) described anti-malarial quality survey(s) in a specific location or region with enough information to yield an estimate of the frequency of poor quality anti-malarials. From the 130 publications, a total of 529 records-surveys are plotted on the AQ Surveyor map. Specific details about the techniques used for analysis, quality result for a specific brand, dosage or pharmaceutical ingredient, were given in 82.3% (107) reports, giving 987 records in the database. The total number of samples included is 9,348, not including reports with more than 680 samples collected in five confiscations and reports with unknown number of samples in 38 reports.

### Geographical and temporal data

**Figure 1:**
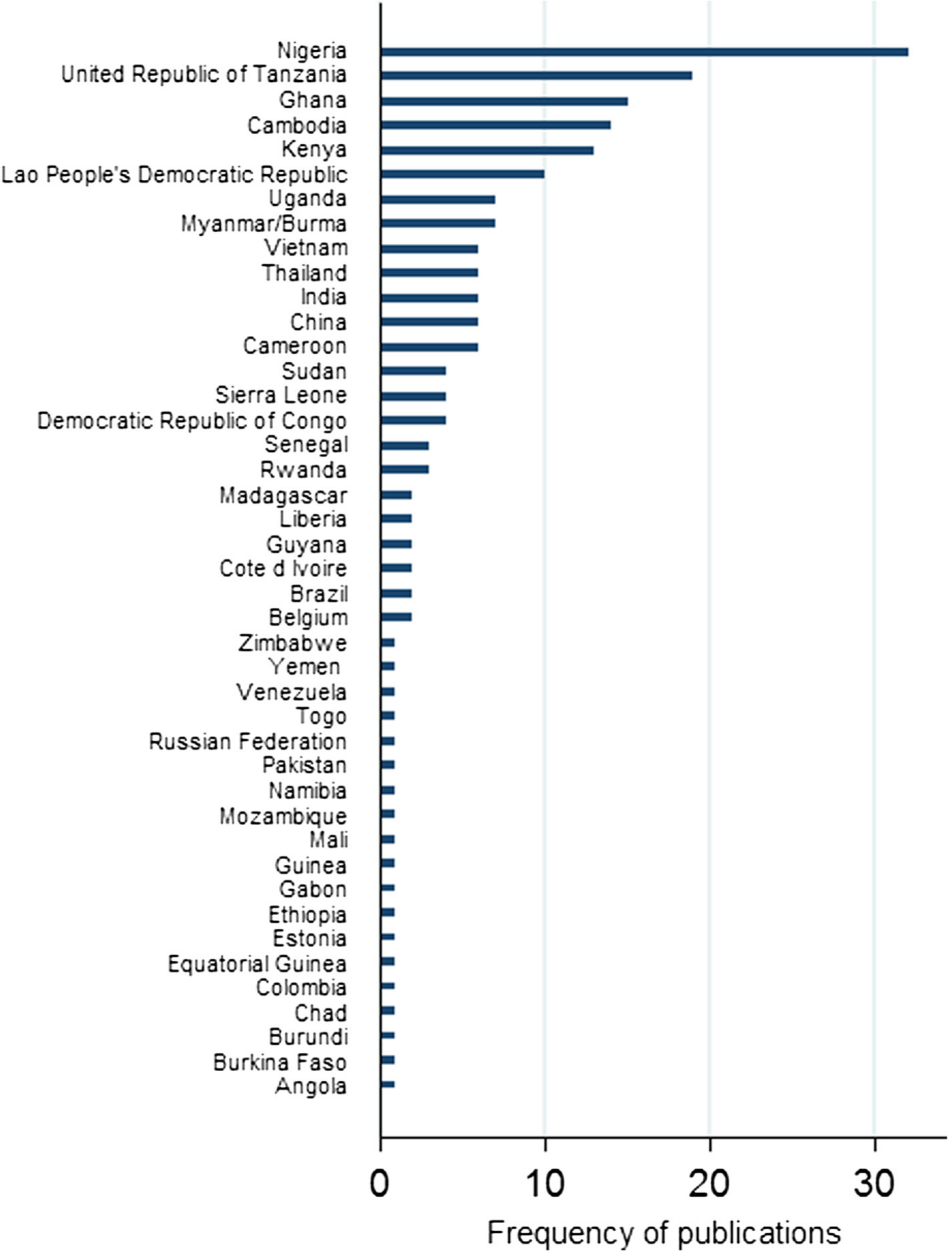
Frequency of reports per country where anti-malarial samples were collected.

Of 104 malaria-endemic countries [1], some published information on anti-malarial quality is publicly available from 41.3% (43), with over half of these (58.1%, 25) only having one or two reports available. Of malarious countries in South and Central America, Africa and Asia, anti-malarial quality data are only available from 19, 61 and 50% countries, respectively. There are no reports from vast swathes of central Africa, such as Zambia. There is only one report each from DRC, Angola and Gabon (that represent 40% of the estimated global burden of malaria); and Sri Lanka, Nepal and Melanesia. There are very few from India, South and Central America and southern Africa. No report was found from the Eastern Mediterranean WHO Region or the six endemic-malaria countries in Europe (Additional file 3). Nigeria has highest number of reports followed by Tanzania, Ghana, Cambodia, Kenya, and the Lao PDR (Laos) (Figure 1). There are only 82 locations listed in the AQ Surveyor, with 36.7% (61) of publications not stating where within countries samples were collected.

**Figure 2:**
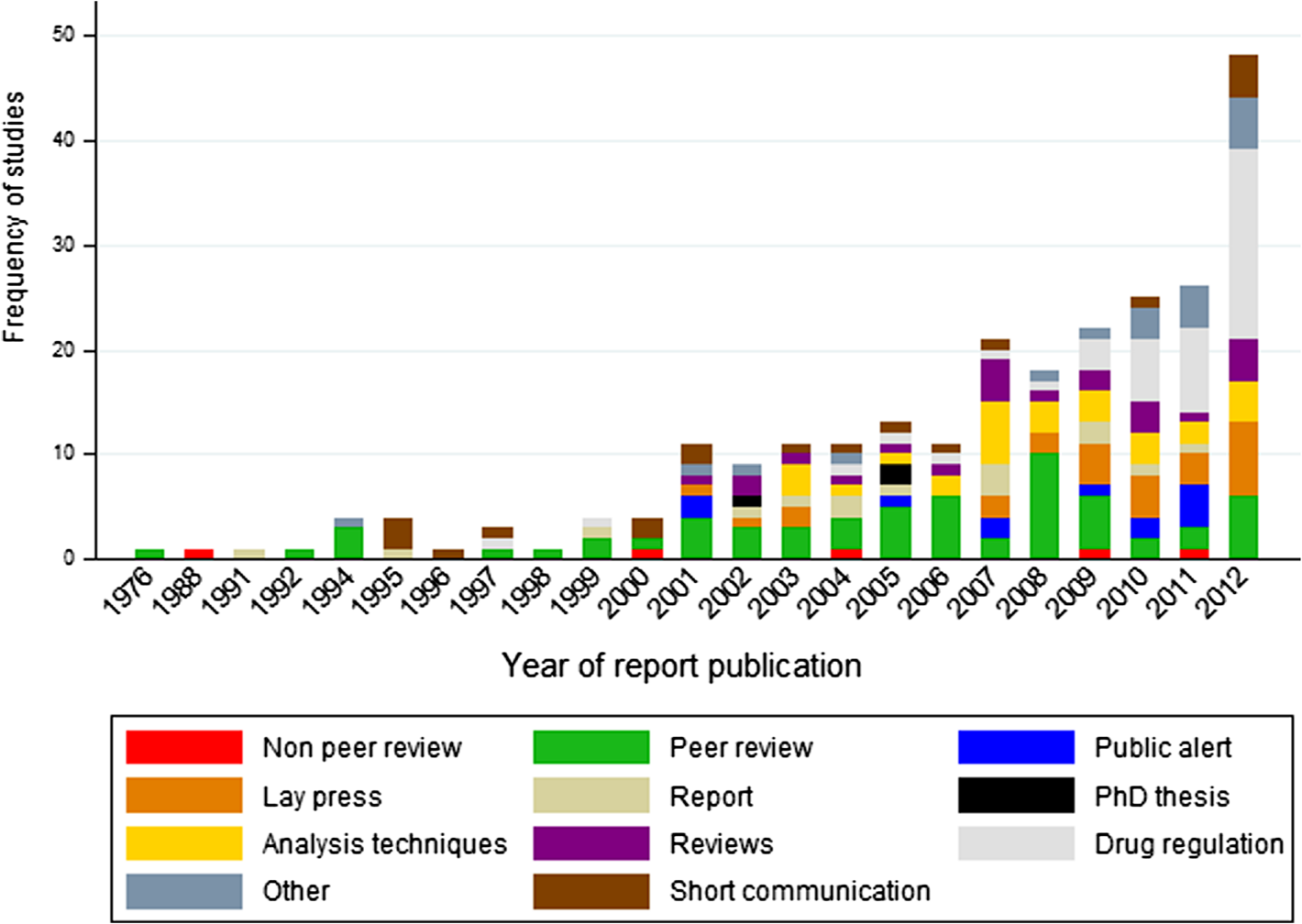
Studies classified by year and by type of publication.

The number of surveys of anti-malarial quality has increased over the last decade (Additional file 4). In the last five years the focus of anti-malarial quality publications appears to have shifted to medicine regulation and chemical analysis techniques, with fewer peer-reviewed primary research articles on anti-malarial quality surveys. Peer-reviewed articles accounted for 24.3% (61) of the 251 publications, with medicine regulation 16.7% (42), analysis techniques 11.2% (28) and lay press 10.4% (26) accounting for the remainder (Figure 2). The median (range) delay between collection and publication was one (zero to 11) years, with 26.7% (39) of publications not stating when the sampling was conducted.

### Sampling and definitions

Of the 130 published reports only 5% (six) included evidence for randomization of sample location selection (Additional file 5) [33-38]. For papers published since the Medicine Quality Assessment Reporting Guidelines (MEDQUARG) [29] for conducting and reporting surveys of medicine quality were published in 2009, only 15.4% (six of 39), describe following them. Of 529 surveys, 32.3% (171) explicitly describe their definitions of counterfeit, falsified, and/or substandard and 20.6% reports (27) used contemporary WHO definitions. Another four reports mentioned the WHO definitions but called the medicines substandard, classifying them according to whether they complied with colour tests, had a different retention time (Rf) value in thin layer chromatography (TLC) or used the terms ‘poor quality’ and ‘substandard’ without conducting packaging analysis and based only on amount of active ingredient.

Of the 130 primary survey research papers, 47.7% (62) stated in which country the chemical analysis was performed, for 32.3% (20) of these, it was performed in high-income, non-malarious countries and in 17.7% (11) the analysis was conducted in WHO prequalified labs.

### Antimalarial Quality surveys

**Figure 3:**
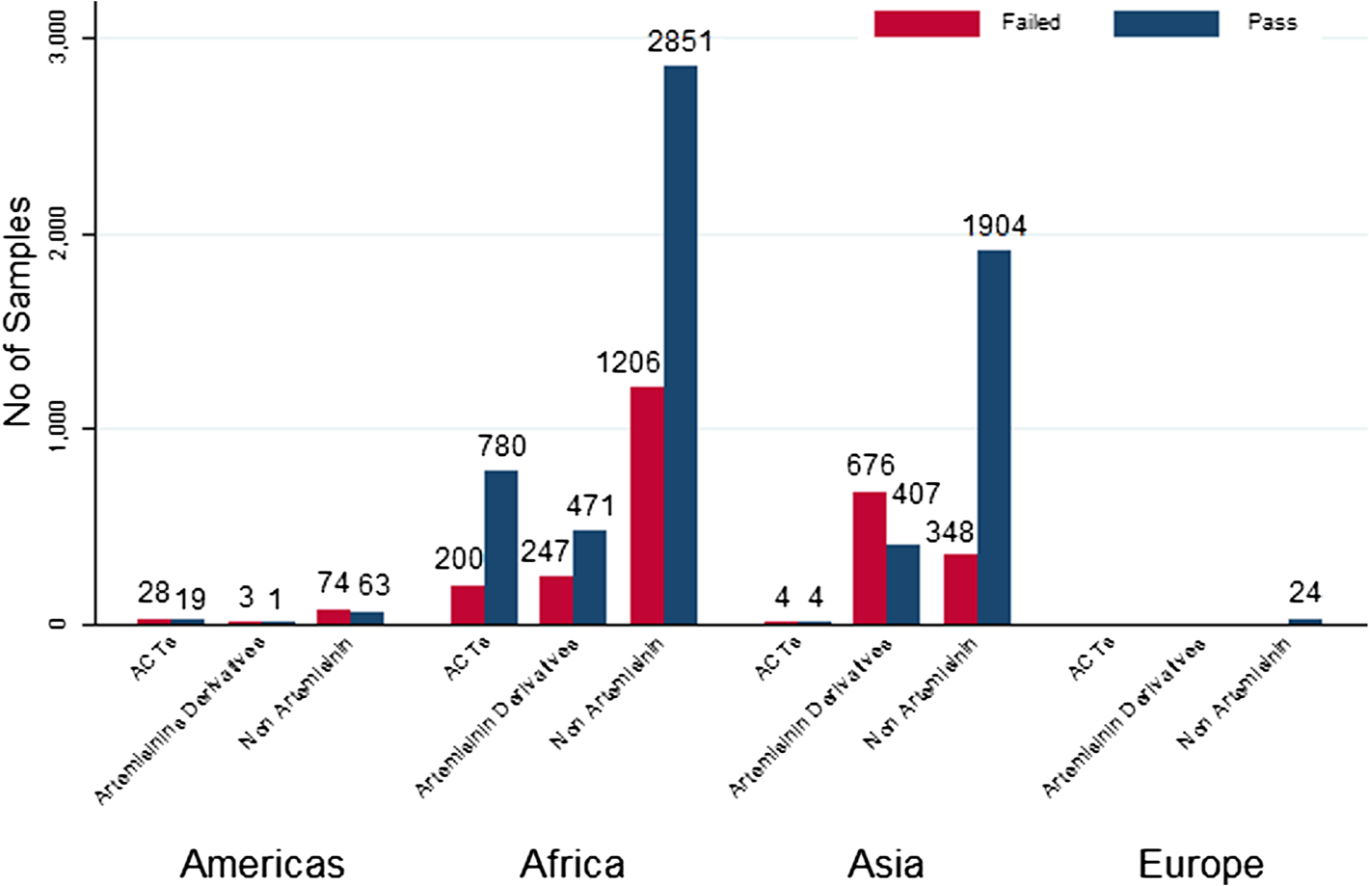
Total number of failing samples classified by anti-malarial category and region.

The majority of the surveys (61.4%, 323) examined the quality of non-artemisinin derivative anti-malarials, especially chloroquine (23.4%, 76) and sulphadoxine-pyrimethamine (SP) (23.1%, 75; see Additional file 6). To date only one report of falsified ACT has been reported in Asia (a seizure in China with medicines for sale in Africa, [5]) but there have been numerous reports from Africa. Figure 3 shows the number of failing samples classified by anti-malarial category and region.

**Figure 4:**
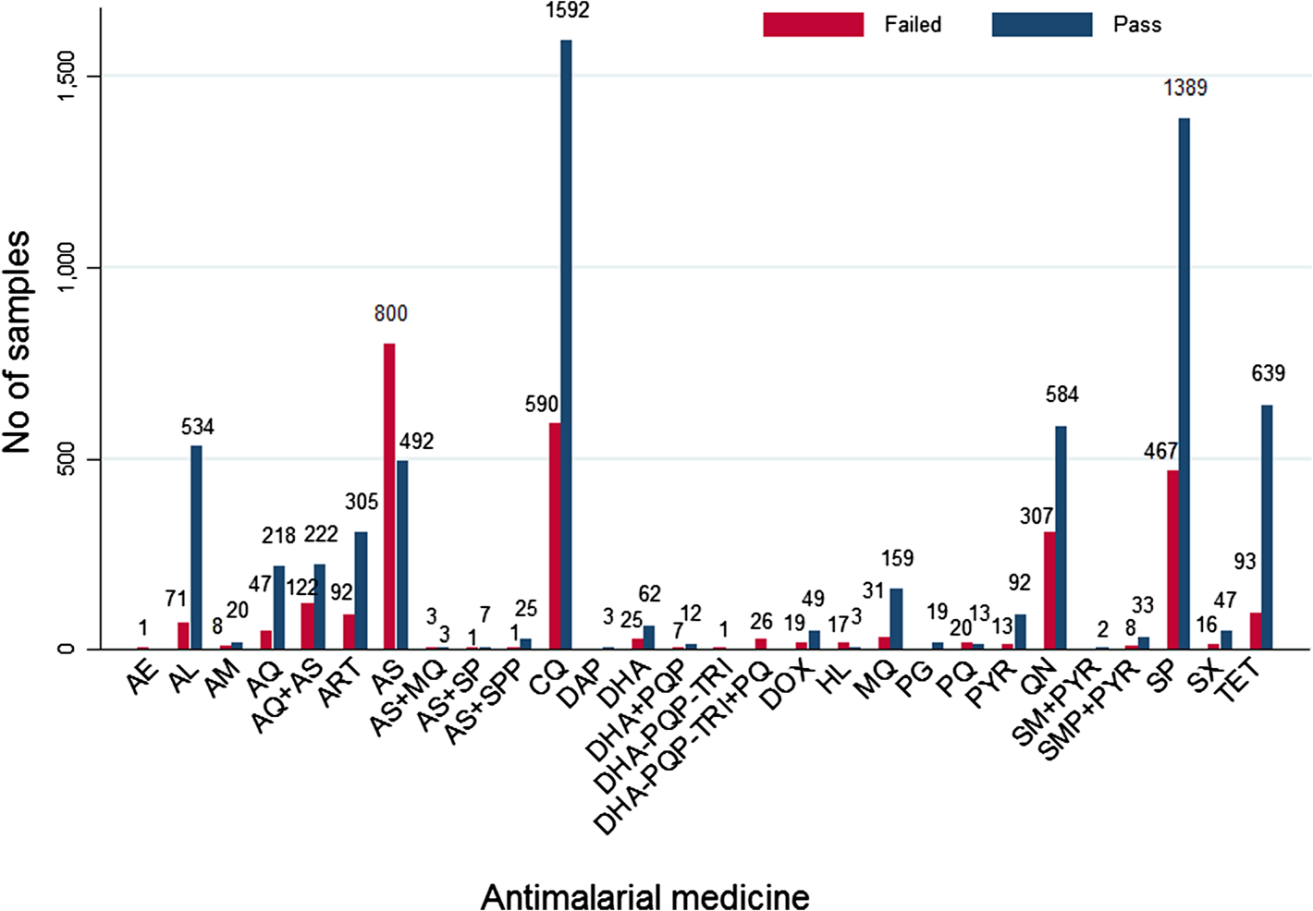
Number of samples that failed and passed a test for quality.

Out of 9,348 samples included in the database, 30.1% (2,813) failed chemical/packaging quality tests. Oral artesunate was the medicine most commonly reported as falsified (with 61.9% of it failing) see Figure 4. Of the 2,813 samples that failed a chemical quality test, 39.3% (1,107) were classified as falsified, 2.3% (66) as substandard and 58.3% (1,640) were classified as poor quality, without evidence to categorize them as either substandard or falsified (Additional file 7).

**Figure 5:**
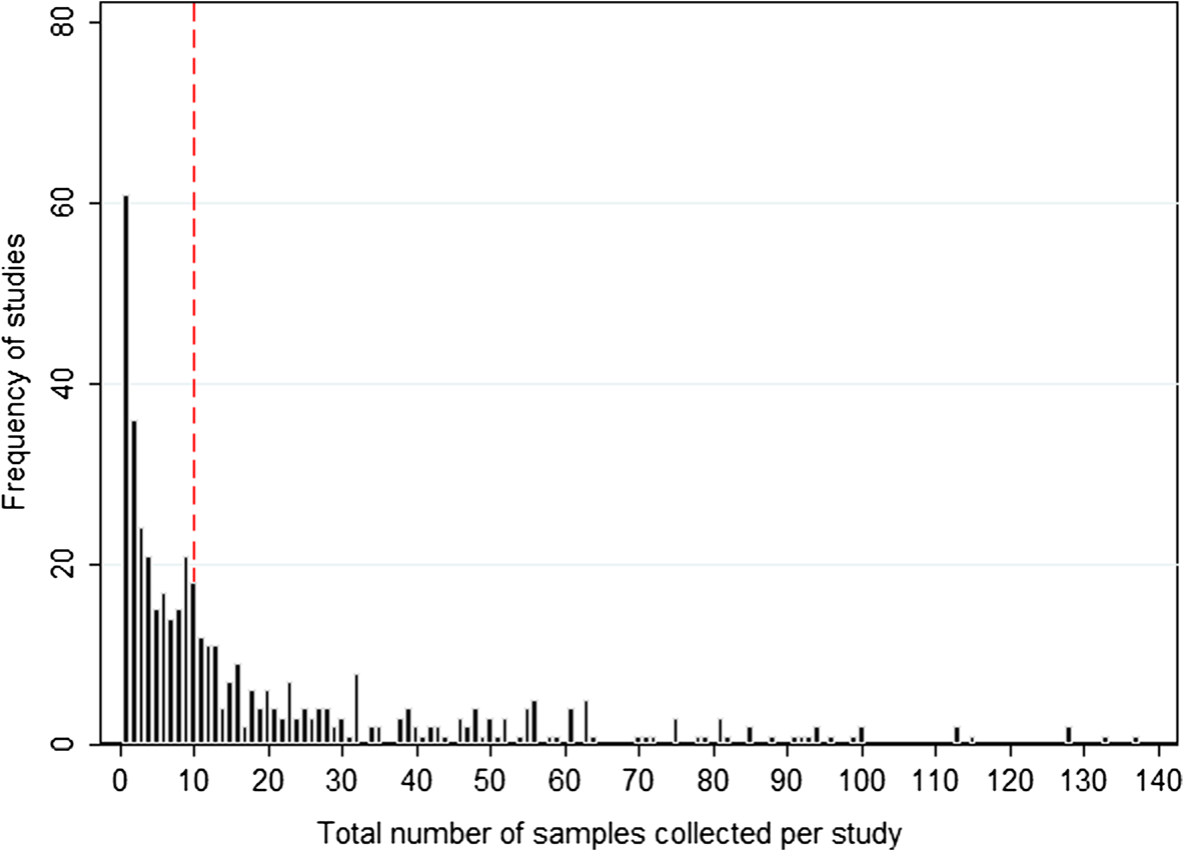
**Number of samples collected per survey.** The red line represents ten samples per study.

An important minority (14.0%, 74) of the 529 surveys did not state the number of samples collected, and 45.7% (242) included less than ten samples per international non-proprietary name (INN) see Figure 5. The median (range) number of samples collected per study was ten (one to 258), excluding reports describing confiscations.

No surveys were found of a significant number of anti-malarials, including atovaquone, cycloguanil, clindamycin, dapsone-pyrimethamine, napthoquine, piperaquine, and parenteral artesunate. Surveys have mainly been conducted on oral artesunate, chloroquine and sulphadoxine-pyrimethamine with a mean number of different anti-malarial INNs collected per study of two, up to a maximum of nine (Additional file 8). As an example, the figure in Additional file 9 gives the different published failure rate results for all the anti-malarials sampled and classified as falsified in Cambodia, a country with many published reports. The numbers vary greatly depending on the number of samples analysed and methodology followed.

Of 130 publications, 45% (59) stated whether the 1,132 failing samples (including samples from one confiscation [39]) had high, low or a different API from that stated on the packaging. Of the failed samples with chemical data, 27.0% (306) had no API detected, 25.7% (291) had high API, 24.7% (280) had low API and 22.5% (255) had wrong API (Additional files 10 and 11). Of samples with different ingredients to those stated on the packaging, 20 different ingredients were found, ranging from aspirin, acetaminophen, mercaptobenzothiazole to soya flour (Table 2).

**Table 2:** Number of samples with active pharmaceutical ingredient found different to what the medicine was labelled as containing

**Active pharmaceutical ingredient found in anti-malarial medicine samples**	**Medicine labeled as**										**References**
	**AS**	**CQ**	**DHA**	**HL**	**MQ**	**QN**	**TET**	**AL**	**SMP + PYR**	**DHA + PQP**	
2-Mercaptobenzothiazole	3										[38]
Acetaminophen	17		1	7					101*		[5,38,40-42]
Artemisinin	4			4							[5,38]
Aspirin		1									[43]
Chloramphenicol	2										[38]
Chloroquine	8					253*					[5,34,38,41,44-46]
Chlorpheniramine		1									[47]
Dimethylfumarate	6										[38]
Erucamide	1										[38]
Erythromycin	7										[38,48]
Erythromycin and Paracetamol (acetaminophen)	8										[49]
Sulphadoxine-pyrimethamine									100*		[50]
Metamizol	5										[38]
Pyrimethamine	8							2			[5,38]
Pyrimethamine and Sulphadiazine								1			[5]
Sildenafil										4	[5]
Soya flour							100*				[50]
Sulphadoxine	12			57600							[38,39]
Sulphadoxine-pyrimethamine	30				1						[51,52]
Sulphamethazine				1							[53]

100* = Unknown number of samples. Missing number of samples from one set of samples of chloroquine, mefloquine, sulphadoxine-pyrimethamine and three set of samples of quinine. Anti-malarial medicines: AS: artesunate, CQ: chloroquine; DHA: dihydroartemisinin; HL: halofantrine; MQ: mefloquine; QN: quinine; TET: tetracycline; AL: artemether-lumefantrine; SMP + PYR: sulphamethoxypyrazine-pyrimethamine; DHA + PQP: dihydroartemisinin-piperaquine.

The types of outlets where medicines were sampled was not specified in 23.8% (126) of the 529 surveys; 2.5% (13) of the surveys stated that they sampled only public-sector outlets, 37.0% (196) included private outlets alone and 28.0% (148) did not specify which results referred to private or public outlets.

### Chemical analysis techniques

Thirty-three different techniques to determine the quality of the medicines was stated in one-hundred and seven publications, including high-performance liquid chromatography (HPLC) 37.4% (40), thin layer chromatography (TLC) 12.1% (13), direct mass spectrometry (MS) 4.6% (five), liquid chromatography–mass spectrometry (LC-MS) 2.8% (three), colorimetry 2.8% (three) and dissolution 24.3% (26). Examination of the packaging was reported in 21.5% (23) publications. For API content analysis technique and interpretation, the US Pharmacopoeia (31.1%, 41) was most commonly used, followed by the British Pharmacopoeia (9.9%, 13), but this vital information was not given in 41.7% (55) of publications.

In 60.2% (53) of reports, details of the manufacturers stated on the sample were not included. In a further 25 reports the country of manufacture was given without the stated manufacturer’s name. Samples labelled, correctly or incorrectly, as made by Guilin Pharmaceutical Co Ltd, China, were the most commonly collected but details of 90 anti-malarial manufacturers from 36 countries, as stated on the packaging, were found in the reports. The alleged country of manufacture was specified in 934 failing samples (of 2,813) and China was the alleged country of manufacture of 35.2% (329) failing samples followed by Pakistan 16.2% (151), India 13.7% (128) and Switzerland 11.8% (110); whether these were the countries of manufacture is unknown.

Fifteen of the 36 (50%) stated countries of manufacture, have stringent regulatory authorities (SRA) that are members/observers/associates of the International Conference on Harmonization of Technical Requirements for Registration of Pharmaceutical for Human Use (ICH) [54].

Of the samples that failed chemical and/or packaging analysis 8.2% (209) were labelled as from a WHO prequalified manufacturer, 0.9% (24) labelled as from a manufacturer with an address within a SRA country and 4.1% (104) labelled both from a WHO prequalification programme (PQP) and from a SRA country. Of the eight WHO prequalified anti-malarial manufacturers [55], products from three manufacturers were found in the database.

## Discussion

The literature review available through the WWARN AQ Surveyor illustrates the alarming scale of poor anti-malarial quality in many malarious countries, and also highlights the major geographical gaps with no published information on the quality of anti-malarials for much of the malarious world and severe problems of data interpretation. The AQ Surveyor is the first freely available global repository compiling all published anti-malarial quality reports from the last 60 years.

### Data gaps

No reports of anti-malarial quality were found for 60.6% (63) of the 104 malaria-endemic countries and 38.6% (17) of the 44 African malarious countries. Although there are clear foci of poor quality anti-malarials, the current global situation remains unclear, poorly documented; and their impact on public health uncertain. The data are insufficient to understand the distribution of ‘hotspots’ of poor anti-malarial quality and the reasons for heterogeneity.

The data suffer from important limitations that are vital to bear in mind in their interpretation. There is likely to be a large unpublished ‘grey literature’ held by MRAs and the pharmaceutical industry that would contain useful information. The pharmaceutical industry, while paying close attention to falsification of their products, are often reluctant to share figures or specific examples [56,57]. The lack of standardization of reporting is also serious limitation. Results are frequently not broken down by country and/or medicine, making it very difficult for individual countries to plan their response unless they are sent data by the authors of reports.

### Difficulties of interpreting the data

Studies are not reported in a consistent manner, hampering comparison with other studies through time and space. The number of samples is commonly not representative and the small number of units tested also limits the interpretation of the results. One third of the reports (29.2%) did not state the number of samples they collected.

Estimates vary greatly depending on the sampling methodology and the technique used, probably greatly influencing reported failure rates. In 13 reports a portable technique was used to determine medicine quality and in eight of those, no confirmation analysis was conducted in a certified quality control laboratory whilst the accuracy of these portable techniques has not been properly evaluated in the field.

Most importantly, randomization, usually considered as the gold standard in estimation of disease prevalence was used very rarely. Without such an objective sampling strategy it will not be possible to obtain confidence intervals, objectively compare between regions or through time or test the effectiveness of interventions [58]. Convenience sampling can be useful for yielding alerts about poor quality medicines but cannot be used reliably to estimate their frequency.

Thirty-four (26%) studies did not report when the collection was conducted and there were long delays, up to seven years, between sample collection and publication.

Ambiguous definitions over what constitutes falsified and substandard medicines make data standardization difficult and 74% of the reports did not state what definition they used. A vital problem is the frequent lack of distinction between falsified and substandard medicines. This distinction requires additional time-consuming packaging analysis but is indispensable, as without these data MRAs do not know if they are dealing with fraud and criminal ‘pharmaceutical’ production or with poorly functioning manufacturing plants, or issues related to medicine storage. How an MRA will respond to reports of poor medicine quality depends on this distinction. This problem is exacerbated by the difficulty of accessing examples of genuine packaging to conduct packaging analysis. Out of the failing samples, 58.3% (1,640) were classified as poor quality. These poor quality anti-malarials tended to have either greater or lesser amounts of API in comparison to that stated on the packaging, suggesting that they are most likely substandard. Quinine and artesunate seem to be the most commonly falsified with wrong API, together with artemether-lumefantrine with no API. SP and chloroquine most commonly had incorrect amounts of API.

A further important issue is that dissolution testing was only included in 24.3% (26) of the chemical analyses, presumably because of the large investments in equipment, funds and laboratory time required. Anti-malarials may have the correct amount of API, but especially for SP, may have very poor dissolution [59]. Recent data suggest that similar problems may affect poor quality ACT [60].

**Figure 6:**
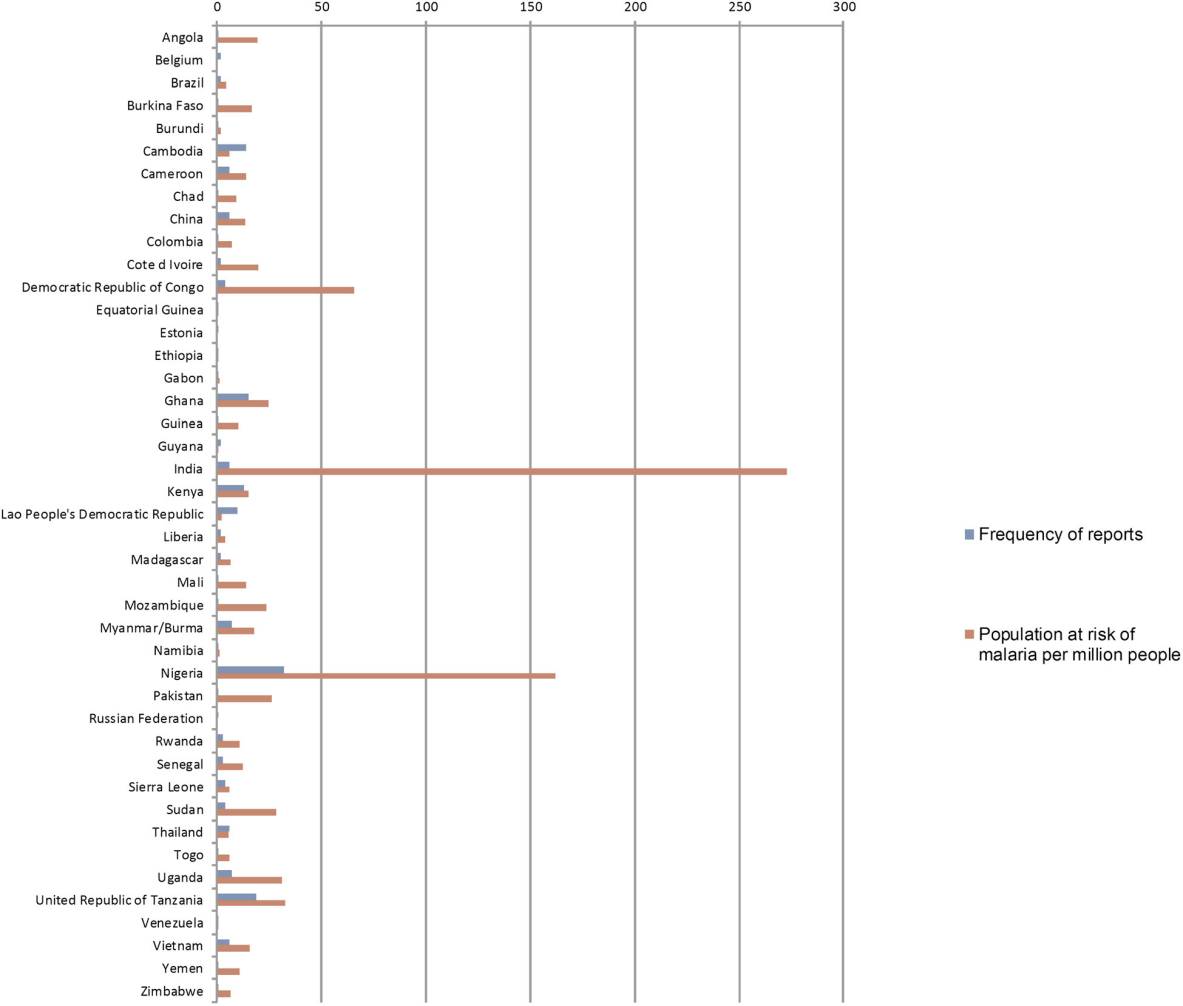
Population at risk of malaria and frequency of reports per country.

More investigation needs to be done in the Americas and the central and southern African regions as there are very few reports from these malarious areas. Six countries: Nigeria, the Democratic Republic of Congo, Burkina Faso, Mozambique, Côte d'Ivoire, and Mali, account for 60%, or 390,000, of estimated global malaria deaths [1], but only one report of anti-malarial quality was found from Burkina Faso and Mali, and two reports for Côte d'Ivoire. There is little confidence in the generalizability of anti-malarial quality for large populations afflicted with malaria. More surveys are needed in populations with high malaria risk (Figure 6). Also, little information is available about the registration status of the manufacturers in endemic countries or about expiry date tampering. Only 13.1% (17) reports stated whether samples were registered in the country of collection and 16.9% stated whether expired samples were found.

Other reviews summarizing the prevalence of poor quality anti-malarials did not have access to a full database of a long period of data in multiple languages [11,61] and gave aggregated estimates of the prevalence of poor quality anti-malarials through space and time without emphasizing the limitations of the data and dangers of over-interpretation. The aggregation of information from sources with variable credibility with equal weight may not be appropriate and this issue needs to be addressed.

Therefore, the data reviewed here *cannot* be summarized as stating that 30.1% of the world’s anti-malarial medicine supply is poor quality. All that can be concluded is, mindful of the above caveats, that 30.1% of anti-malarials tested over 67 years were poor quality and that this is an important public health problem. Enormous quantities of anti-malarials are consumed in comparison to the number of samples with published quality data. In Africa, an estimated 200-400 million courses of anti-malarial treatment are used per year with an additional 100 million courses elsewhere [62]. The number of ACT treatment courses delivered globally to the public and private sectors increased from 11 million in 2005 to 278 million in 2011 [1]. However, information of the quality of 9,348 samples and 1,034 ACT were only found since 1946.

### ‘Talk only, no action’

Despite evidence that poor quality anti-malarials are focally common and are bad for patients, there have been few interventions and the subject has been dominated by discussions with little action. The efforts put into addressing the quality of medicines have had little tangible impact, in comparison to the size of the problem, because the programmes have mostly been slow, under-funded and fragmented. Many parallel interventions are needed [3,5,11,16,19,63]. The controversy over definitions has disabled much that could be done and the use of the unwieldy term ‘substandard/spurious/falsely labelled/falsified/counterfeit medical products (SSFFCs)’ has not helped. The international community should act with much greater speed and focus to build interventions to safeguard global medicine supply (Newton *et al*. submitted).

The newly created Member State Mechanism on SSFFCs should rationalize this term into definitions that are accessible and accurate and aid public health [16,17], help to focus on the development and maintenance of up-to-date international tools, guidelines and standards, identify major needs and challenges and make policy recommendations to strengthen national and regional capacities [64-68]. The provision of free or subsidized ACT through the Global Fund, President’s Malaria Initiative (PMI), the World Bank and other major donors has probably had a large impact on improving the quality of anti-malarials that patients take in the public sector, although it was not designed with this primary aim in mind [62]. The WHO Rapid Alert System [69] should allow a better coordinated sharing of information and help fill in the many gaps. However, more data from countries with high malaria incidence but few data points on anti-malarial quality are urgently needed. Similarly, the pharmaceutical industry, both innovative and generic, should have a legal duty to report suspicions of poor quality medicines to key stakeholders such as MRAs and WHO [56]. A major problem is that there are very few laboratories in the malarious world able to accurately analyse the quality of anti-malarials – there are only three countries in sub-Saharan Africa and five in Southeast Asia with WHO prequalified laboratories [70]. Many of the current problems have arisen as a consequence of the lack of investment in MRAs in low- and middle-income countries, by national and international organizations, to allow them to effectively regulate and police the medicine supply. Of 100 primary survey research papers not performed by MRAs, only 26% (26) mentioned that they informed the MRA. Appropriate interventions to support MRAs are urgently needed.

## Conclusions

The data available for assessing the frequency of poor quality anti-malarials and their public health impact are of poor quality but suggest that there are severe problems at least in some important foci with high malaria burden. Poor-quality antimalarials that contain sub-therapeutic amounts of active ingredient increase the risk of drug resistance and may put at risk current therapeutic control strategies. There is an immediate need for standardizing sampling and assay methods and achieve consensus in defining different types of poor quality medicines.

In a social and economic landscape where 30% of the world´s MRAs do not have functional capacity and most developing countries do not have national laboratories [71], inspectors are not able to objectively screen for suspect medicines. In order to properly assess the quality of medicines, it needs to be ensured that such techniques are accessible in lower income countries and, as has happened in wealthy countries, empowering routine inspection must be a much higher public health priority.

### Ethics statement

An ethics statement was not required for this work.

## Abbreviations

WWARN: Worldwide Antimalarial Resistance Network; APIs: Active pharmaceutical ingredients; IP: Intellectual property; GPHF-Minilab®: Global Pharma Health Fund; ACT: Artemisinin combination therapy; WHO: World Health Organization; USP: United States Pharmacopeia; MRA: Medicines Regulatory Agencies; AQ Surveyor: Antimalarial quality surveyor; NMCPs: National malaria control programmes; INN: International non-proprietary name; MEDQUARG: Medicine quality assessment reporting guidelines; Rf: Retention time; TLC: Thin layer chromatography; HPLC: High-performance liquid chromatography; MS: Mass spectrometry; LC-MS: Liquid chromatography–mass spectrometry; SRA: Stringent regulatory authorities; ICH: International Conference on Harmonization of Technical Requirements for Registration of Pharmaceutical for Human Use; PQP: WHO prequalification programme; SP: Sulphadoxine-pyrimethamine; SSFFCs: Substandard/spurious/falsely labelled/falsified/counterfeit medical products; PMI: President’s malaria initiative.

## Competing interests

The authors have declared that they have no competing interests.

## Authors’ contributions

PTE conducted the literature search, designed the database and the map, conducted the analysis and wrote the first draft. PNN designed the project, supported the literature search and provided scientific support. PG, FF and MG provided scientific support. All the authors read and approved the final manuscript.

## Authors’ information

Patricia Tabernero and Paul N. Newton: Worldwide Antimalarial Resistance Network (WWARN), Churchill Hospital, University of Oxford, Oxford, UK.

## Supplementary Material

Additional file 1 Prisma chart. Click here for file

Additional file 2 WHO definitions for falsified and substandard medicines. Click here for file

Additional file 3 Type of publication per region. Click here for file

Additional file 4 **Number of surveys by medicine category and year of publication.** (A total of 529 surveys are included in 130 reports). Click here for file

Additional file 5 Type of sampling methodology followed in the reports. Click here for file

Additional file 6 Frequency of surveys and percent given by anti-malarial category classification. Click here for file

Additional file 7 **Medicine quality category classification of failing samples.** (Poor quality medicines may include many substandard medicines as this distinction cannot be reliably made without reference to packaging). Click here for file

Additional file 8 Number of samples per medicine surveyed. Click here for file

Additional file 9 Failure rate obtained from the anti-malarials classified as falsified in Cambodia. Click here for file

Additional file 10 Description of failing samples and amount of active ingredient found. Click here for file

Additional file 11 **Description of failing samples and amount of active ingredient found in falsified, substandard and poor quality medicines.** Samples that failed chemical assays, but without detection of wrong active ingredients and without packaging analysis, are classified as poor quality and not as falsified or substandard as this distinction cannot be reliably made without reference to the packaging. Poor quality medicines may therefore include many substandard medicines. Click here for file
