# Supplementary figures and images for: Mind the gaps - the epidemiology of poor-quality anti-malarials in the malarious world - analysis of the WorldWide Antimalarial Resistance Network database
Source: Malar J. 2014 Apr 8;13:139. doi: 10.1186/1475-2875-13-139 (PMC4021408; doi:10.1186/1475-2875-13-139)

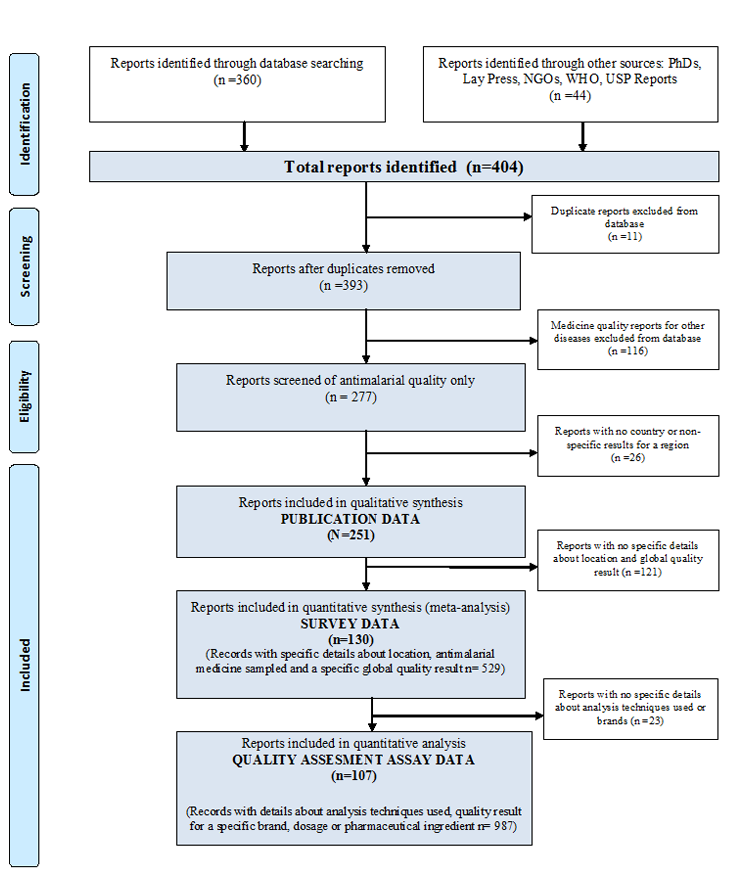

Supplement: Additional file 1 — Prisma chart. [file 1475-2875-13-139-S1.tiff]

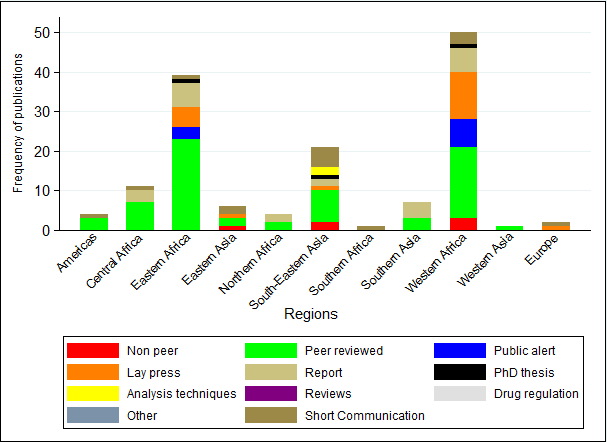

Supplement: Additional file 3 — Type of publication per region. [file 1475-2875-13-139-S3.tiff]

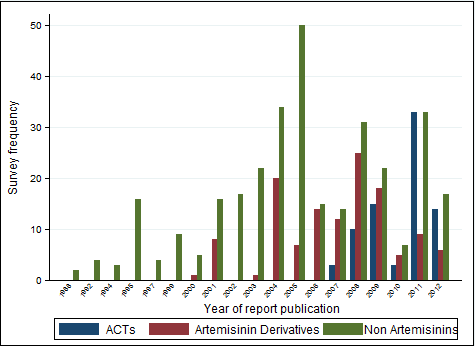

Supplement: Additional file 4 — Number of surveys by medicine category and year of publication. (A total of 529 surveys are included in 130 reports). [file 1475-2875-13-139-S4.tiff]

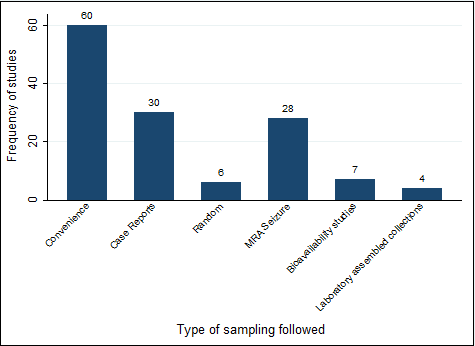

Supplement: Additional file 5 — Type of sampling methodology followed in the reports. [file 1475-2875-13-139-S5.tiff]

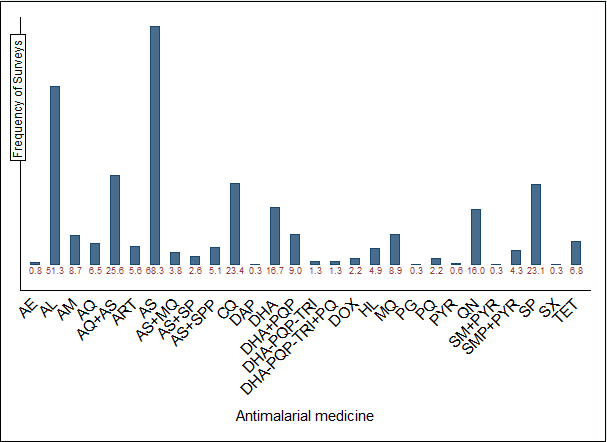

Supplement: Additional file 6 — Frequency of surveys and percent given by anti-malarial category classification. [file 1475-2875-13-139-S6.tiff]

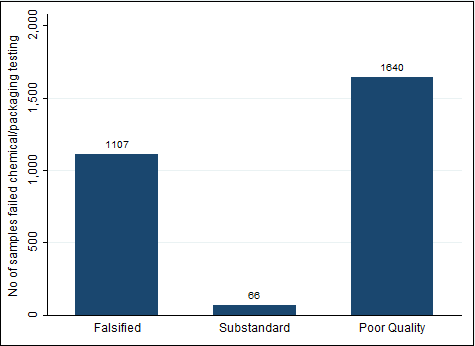

Supplement: Additional file 7 — Medicine quality category classification of failing samples. (Poor quality medicines may include many substandard medicines as this distinction cannot be reliably made without reference to packaging). [file 1475-2875-13-139-S7.tiff]

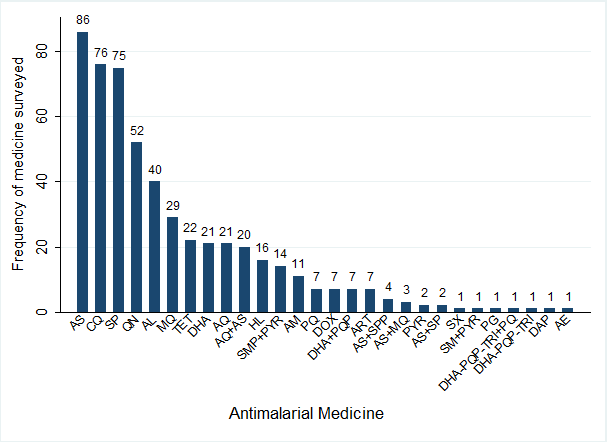

Supplement: Additional file 8 — Number of samples per medicine surveyed. [file 1475-2875-13-139-S8.tiff]

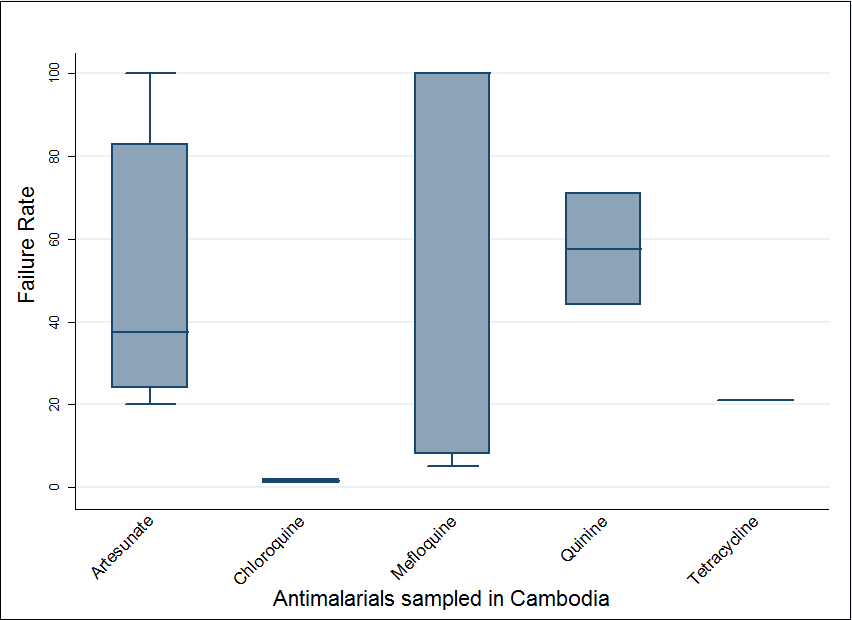

Supplement: Additional file 9 — Failure rate obtained from the anti-malarials classified as falsified in Cambodia. [file 1475-2875-13-139-S9.tiff]

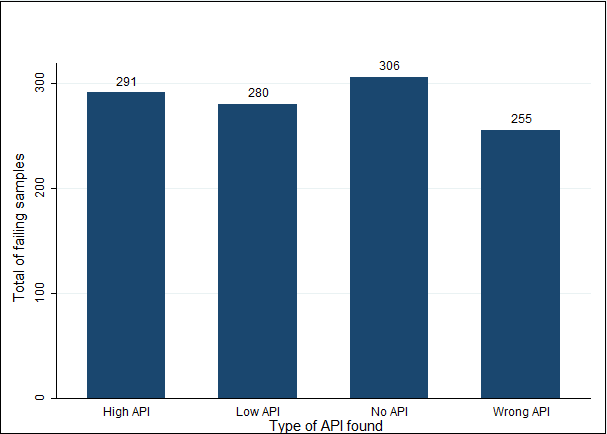

Supplement: Additional file 10 — Description of failing samples and amount of active ingredient found. [file 1475-2875-13-139-S10.tiff]

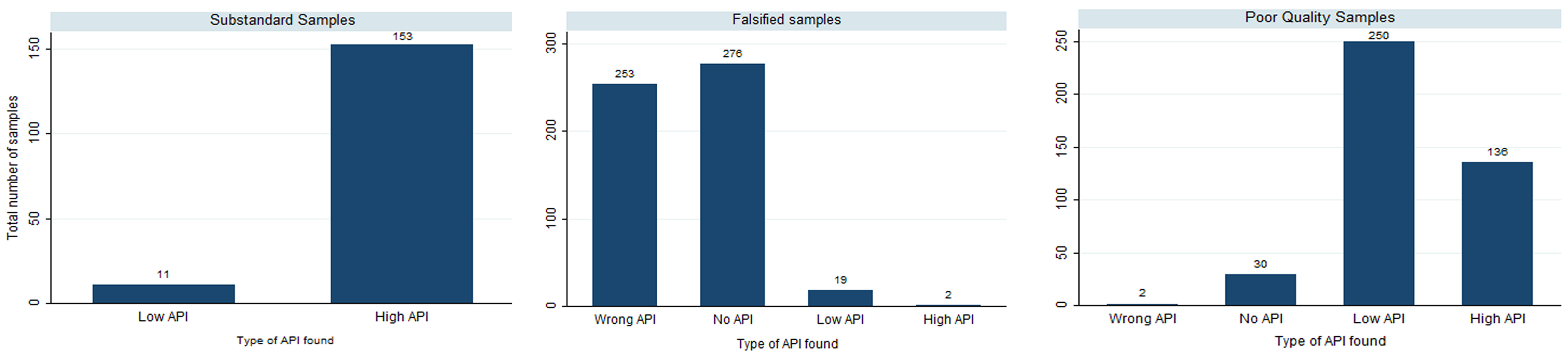

Supplement: Additional file 11 — Description of failing samples and amount of active ingredient found in falsified, substandard and poor quality medicines. Samples that failed chemical assays, but without detection of wrong active ingredients and without packaging analysis, are classified as poor quality and not as falsified or substandard as this distinction cannot be reliably made without reference to the packaging. Poor quality medicines may therefore include many substandard medicines. [file 1475-2875-13-139-S11.png]
