# Supplementary material for: Mind the gaps - the epidemiology of poor-quality anti-malarials in the malarious world - analysis of the WorldWide Antimalarial Resistance Network database
Source: Malar J. 2014 Apr 8;13:139. doi: 10.1186/1475-2875-13-139 (PMC4021408; doi:10.1186/1475-2875-13-139)
Supplement: Additional file 2 — WHO definitions for falsified and substandard medicines. [file 1475-2875-13-139-S2.pdf]

- Spurious/falsely labelled/falsified/counterfeit (SFFC) medicines are medicines that are deliberately and fraudulently mislabelled with respect to identity and/or source
- Substandard medicines (also called out of specification (OOS) products) are genuine medicines produced by manufacturers' authorized by the MRA which do not meet quality specifications set for them by national standards

WHO Fact sheet N°275, May 2012
